# E-learning platforms in ideological and political education at universities: students’ motivation and learning performance

**DOI:** 10.1186/s12909-024-05572-2

**Published:** 2024-06-06

**Authors:** Huabing Yang

**Affiliations:** School of Marxism, Hunan City University, No.518, Yingbin East Road, Yiyang, 413000 China

**Keywords:** Academic progress, Digitalization of education, E-learning platform MOODLE, Educational motivation, Ideology, Policy

## Abstract

**Background:**

The study aimed to examine the impact of using the MOODLE e-learning platform in ideological and political education on Chinese students’ motivation and academic performance.

**Methods:**

The study involved 447 students from China-based universities (the experimental group − 232 students who studied using electronic educational platforms, and the control group − 215 students who used no digital technologies in their learning). The following methods were used: Measuring the need to achieve success among students; T. I. Ilyina’s method for studying motivation to study at university; Method for studying student success motivation; Method for studying the motives of students’ educational activities; Method for determining the main motives for choosing a profession (E. M. Pavlyutenkov); Motivation of learning activities: Levels and types (I. S. Dombrovskaya). Students’ academic performance was assessed by testing in the studied disciplines at the beginning and end of the study.

**Results:**

As a result, the significance of the motivational component in achieving the success of ideological and political education and the impact on students’ motivation to use e-learning platforms is theoretically substantiated.

**Conclusions:**

It has been confirmed that using e-learning platforms in ideological and political education helps increase student motivation and academic performance.

## Introduction

Political activity in today’s global world is essential. It is interconnected with socio-economic development, scientific and technological, and information-technological progress. Education is recognized as the foundation of China’s national development and progress and is a priority of its national policy [1]. Ideological and political education in China’s universities is closely related to the process of moral education of students, the formation of their ideals and beliefs as the results of a spiritual search [2]. Ideology is understood as a determinant of policy. Introducing machine learning based on artificial intelligence into ideological and political education increases teaching effectiveness and improves student performance more than traditional teaching [3].

As a means of distance learning, the Internet provides almost unlimited access to high-quality educational resources [1]. The rapid development of networked educational technologies necessitates continuous improvement of ideological and political education based on them as the most critical aspect of education for the builders of a socialist society [1]. Accordingly, ensuring a high ideological and political education level is essential to the education system. Its development prospects are considered in the context of the use of artificial intelligence and e-learning platforms, which places high demands on the quality of building an educational information system and network ideological and political education platform [4–6].

Ideological and political education is a theoretical discipline taught in all universities and colleges in China, which determines the relevance of the digitalization of the educational process based on the algorithm for applying e-learning platforms [7]. The need to provide high-quality modern ideological and political education is a factor that stimulates the development and implementation of innovative technologies in education and their continuous improvement [8, 9]. This trend requires students and teachers to have a high level of adaptability to new technological conditions of the educational process [10].

Data from scientific literature sources indicate the positive role of intrinsic and extrinsic learning motivation in the acquisition of political literacy by students, which improves their academic performance in ideological and political courses and general subjects [2]. The use of e-learning platforms in ideological and political education allows for continuous improvement by providing opportunities for sharing experiences, evaluating the motivational and communicative aspects of the educational process, analyzing academic performance, and selecting optimal learning models [11].

Thus, information technology is a principal tool and a good resource for modern education [12]. The use of artificial intelligence and e-learning platforms in ideological and political education is a promising direction, the development of which is significant for the Chinese education system, aimed at educating a new generation of builders of a socialist society [13], which determines the relevance of this research.

*The scientific novelty of the study* lies in the theoretical substantiation of the importance of the motivational component in achieving the success of ideological and political education and the impact on students’ motivation to use e-learning platforms.

*The present findings* help improve the effectiveness of ideological and political education in China-based universities, which is also of interest to the international scientific and pedagogical community in a global multicultural educational environment.

## Literature review

### Using e-learning platforms as a promising direction for developing the education system

Teaching and learning in an online environment ensured the continuity of the educational process during the COVID-19 pandemic, demonstrating the promise and potential of using e-learning platforms to expand educational opportunities [14]. The adaptation of the education sector to technological progress, the spread of Internet technologies, cloud and wireless technologies with 4G and 5G support occurs through the replacement of traditional audiences with online audiences, electronic classes and video conferencing, and the use of educational applications and websites [15].

Modernization of learning by transforming traditional classrooms into digital educational spaces with visualization and gamification, which involves the use of game rules for educational purposes, enhances the interaction between teacher and student, improves the quality and accessibility of educational materials, and also stimulates students to work independently [7]. This is due to the fact that the game format makes it possible to make educational tasks more interesting, and understanding the educational material with this approach becomes easier [7]. Electronic educational platforms based on cloud computing technology are a practical resource for teaching and learning software, meeting the diverse needs of education informatization through the creation of digital campuses, which optimizes the management of the educational process by improving the interaction between all its participants [16].

Thus, e-learning platforms have created conditions for the effective accumulation and sharing of knowledge in the online educational environment [17].

### The impact of digitalization of the educational process on student motivation

The transformation of educational practices based on innovative online digital technologies aims to ensure the formation of students’ competencies as a necessary condition for their personal and professional development [18]. Students’ motivation for learning determines their achievement of educational goals. Accordingly, the development of e-learning platforms is carried out, taking into account the possibility of using them as a mechanism that perceives and stimulates the motivational state of students [19]. Electronic educational platforms are an information space that serves as an intermediary between teacher and student, thereby increasing the effectiveness of online learning. Meanwhile, researchers [20] cite a significant dropout rate of students because of their lack of motivation as one of the major problems with the use of e-learning platforms. The introduction of gamification can only partially address this issue, as it does not impact students’ intrinsic motivation, despite the increased involvement, unless individual learning styles are considered [20].

It is recommended to use gamification in combination with “flipped classrooms,” which primarily aim to stimulate learning progress for students lagging to increase the motivational component of education [21]. The flipped classroom is a type of blended learning that combines traditional classroom teaching and e-learning elements using special information technologies. The flipped classroom involves a shift in emphasis to independent study of educational material, with its subsequent practical consolidation during classroom lessons. Many researchers [22–24] argue that such training is an effective way to increase students’ motivation and engagement, develop their ability to learn independently and achieve better academic results. The use of educational platforms and virtual classrooms is believed to have become a technological breakthrough in learning. It has shown the positive impact of educational applications on students’ academic performance, primarily due to the activation of the motivational component of the educational process [25].

### Opportunities and prospects for the use of e-learning platforms in ideological and political education

Integrating the “Internet plus” model into all aspects of human life contributes to digital socialization and innovations such as cloud computing, the Internet of Things (IoT), big data, and blockchain technology in education. It is seen as a progressive approach to ideological and political education [26]. Network electronic educational platforms make it possible to correlate the construction of ideological and political education with students’ characteristics and access to educational resources. They ensure not only the transfer of theoretical knowledge but create conditions for moral development and the formation of student value orientations [27].

The reform of ideological and political education based on the application of digital technologies, electronic learning platforms and mobile phones as a carrier of artificial intelligence has many advantages compared to traditional education due to the possibility of greater interactivity via the organization of discussion forums, electronic audiences, blogs, chats, educational applications [28]. Electronic educational platforms and websites create the conditions for building a high-tech ideological and political education system in universities and colleges [15]. The creation of an electronic data exchange platform in the process of ideological and political education enables supporting research in related fields, exchanging valuable statistical data and improving their use, as well as increasing the content and practical orientation of education [17, 29, 30].

Electronic educational platforms ensure the orientation of ideological and political learning to meet the needs of students and teachers [16]. The application of artificial intelligence and wireless networks contributes to the strategic direction and tactical goals of ideological and political learning by providing opportunities for monitoring the learning process of students, their participation in entrepreneurial activities, and the control of academic performance [31]. Developing an ideological and political framework for the curriculum is a principal aspect of ideological and political education. Using MVC architecture and MVC (Model View Controller) architectural pattern to develop a web application allows working with large databases. It also provides control over the learning process of each student, which ensures the individualization of the educational process [32].

Digital educational technologies provide an opportunity for its individualization by building a student’s portrait to improve ideological and political learning. Thus, the BILDES model allows for obtaining several general characteristics and evaluating the student’s learning style, analyzing their features, communicative qualities and learning style, and determining the motivational orientation and emotional attitude to learning [33]. This approach contributes to the early detection of possible problems in assimilating ideological and political education courses and provides timely intervention to solve them [33].

Building an ideological and political education system based on a hybrid approach, combining online and offline classes is more effective, providing more opportunities than traditional offline learning [34]. The use of information platforms maximizes the integration of ideological and political learning into all components of the educational process through online Internet resources, the possibility of online discussions, and increased individualization and interactivity of classes [12]. The development of university ideological and political multimedia network teaching and its implementation contribute to students’ better assimilation of teaching materials and improve their academic performance [35]. Technologies for recognizing students’ facial expressions during classes are proposed, which help monitor their psychological state in real-time and make appropriate adjustments to the conduct of classes [36].

Education is closely related to culture, traditions, ideology and politics, which determines the importance of ideological and political education. An important factor in political education is to ensure its motivational aspect, the incentive mechanism [37]. It is proposed to use the Deep Learning-Based Innovative Ideological Behavior Education Model (DL-IIBEM), which focuses on stimulating the exchange of information, improving the interactivity of the educational process, and increasing the efficiency of the platform. The goal is to teach innovative ideological behavior using electronic educational media [37, 38].

Artificial intelligence technologies’ most promising uses in ideological and policy education are intelligent wireless neural network capabilities to integrate online and offline teaching and learning [10] and the construction of smartphone-based learning [15]. The emergence and development of network multimedia using mobile devices have significantly impacted modern students’ thinking, worldview and lifestyle, broadly defining value orientations and ideological attitudes. They must be considered during students’ ideological and political education and forming their personal and professional competencies, worldviews, and beliefs [39].

### Problem statement

China’s education policy aims to improve the quality of education based on the informatization of the education process, the integration of big data into the education process, the improvement of information literacy of teachers and students, and the improvement of the quality assessment system of teaching and learning [40]. Information learning platforms help students better learn the value of innovative and creative thinking, contributing to the comprehensive development of each student’s personality [12]. However, as the results of the analysis of scientific literature sources showed, one of the problems of using artificial intelligence for ideological and political education is the lack of an emotional component, which can negatively affect the motivational component of learning [36]. The search for a solution to this problem led to the present study.

*The purpose of the study* was to examine the impact of using the e-learning platform MOODLE in ideological and political education on the motivation and academic performance of Chinese students.

#### Research objectives

(1) analyzing the data of scientific literature sources on the use of e-learning platforms in ideological and political education; (2) conducting an experimental study of the dynamics of students’ motivation and academic performance in ideological and political education using e-learning platforms.

*The research hypothesis* builds on the assumption that using e-learning platforms in ideological and political education increases student motivation and improves academic performance.

## Methods and materials

### Research design

The study was designed to be conducted in stages. In the first stage, the relevance of the research topic was determined, and the sources of scientific literature on the problem of digitization of education and the use of e-learning platforms in ideological and political education were analyzed.

In addition, the problematic aspects of providing the motivational component of ideological and political education using e-learning platforms are identified, and the purpose and objectives of the study are formulated. The second stage envisaged the formation of a sample of respondents and selecting valid psychodiagnostic methods to study the dynamics of students’ motivation in ideological and political education using e-learning platforms. The study’s third stage consisted of conducting the experiment, statistical processing and analyzing its results. The final, fourth stage of the study involved discussing the results obtained, formulating conclusions, and determining the prospects for further research.

### Research procedure

*The sample of respondents* was selected by a simple random sampling method from undergraduate students who are receiving ideological and political education at universities in China. *The data collection process* was carried out in the form of testing using a Google form. Initially, 500 questionnaires were sent out, but 38 students refused to participate in the study at various stages, while 15 respondents failed to answer all the test items. Thus, 447 respondents (265 boys and 182 girls, average age 22.4 years) took part in the survey, of which the experimental group consisted of 232 students who studied using the electronic educational platform MOODLE (Modular Object-Oriented Dynamic Learning Environment). The experiment was carried out for a month, during which the remaining 215 respondents who made up the control group used no digital technologies in their learning. Testing was performed twice: at the experiment’s beginning and end.

### Description of the educational platform

The MOODLE educational platform is a modular, object-oriented learning environment that provides teachers and students with convenient and diverse e-learning tools in online and offline formats that can be used both in the classroom and for self-study at home. This platform has the full range of functions that are necessary to ensure comfortable and high-quality learning. It creates conditions for convenient and effective interaction between teachers and students, makes it easy to manage the learning process and makes the teaching process interesting and unique.

### Research methods

*The study used the following psychodiagnostic techniques*, adapted and validated for Chinese respondents:

- Measuring the need to achieve success among students [41] - a technique consisting of 22 statements, agreement or disagreement with which allows to determine the level of motivation to achieve success in learning activities (1–7 points - low, 8–14 points - average, 15–22 points - high level of motivation for success).
- T. I. Ilyina’s method for studying motivation to study at university [42] is a questionnaire of 50 questions. The answers allow determining the hierarchy of motives for studying at the university: “Acquisition of knowledge” (maximum on a scale of − 12.6 points), “Mastering a profession” (maximum − 10 points), and “Getting a diploma” (maximum − 10 points). The predominance of motives on the scales “Acquiring knowledge” and “Mastering a profession” testifies to the adequacy of the student’s choice of future profession and satisfaction with the learning process.
- Method for studying student success motivation [43]. It means a questionnaire consisting of 36 statements, the answers evaluated on a 5-point Likert scale (Strongly disagree – 1 point, Somewhat disagree – 2 points, Neither agree nor disagree – 3 points, Somewhat agree – 4 points, Strongly agree – 5 points). At the same time, the maximum severity of each parameter is 20 points. With the help of this technique, the exteriorization of success is determined (success as a material standard of living, success-luck, success-recognition, and success-power). Moreover, its internalization is identified (success as a result of one’s activity, personal success, success as a mental state, success as overcoming obstacles, and success-vocation).
- Method for studying the motives of students’ educational activities [44] consists of selecting and ranking the five most significant reasons from the proposed options. In particular, they are as follows: become a highly qualified specialist; get a university degree; successfully continue education in subsequent courses; study well and pass exams with mostly “excellent” and “good” marks; constantly get a scholarship; acquire profound knowledge; be prepared for following classes; be consistent in studies of disciplines; keep up with the academic performance of peers; acquire the skills for a successful career; meet teaching standards; be respected by teachers; set an excellent example for other peers; be praised by parents and others; avoid condemnation and punishment for poor academic performance; enjoy intellectual satisfaction.
- Method for determining the main motives for choosing a profession (E. M. Pavlyutenkov) [45] represents a questionnaire of 18 judgments about the career, expressing nine groups of motives (social, moral, aesthetic, cognitive, creative, informative, material, prestigious, utility). It is evaluated on a 5-point Likert scale (Disagree – 1, Somewhat disagree – 2, Neither agree nor disagree – 0, Somewhat agree – 4, Agree – 5).
- Motivation of learning activities: Levels and types (I. S. Dombrovskaya) [46] consists of 30 statements, the answers to which are evaluated on a 5-point Likert scale (Never – 0, Very rarely – 1, Sometimes – 2, Almost always – 3, Always – 4). It allows identifying the levels of cognitive and social motivation development (broad and narrow cognitive and social motives) (low level of motivation for learning activities – below 2 points, average level – 2–3 points, a high degree of severity of the level or type of motivation – over 3 points).

Students’ academic performance in the experimental and control groups was assessed by testing in disciplines studied at the beginning and end of the research and comparing the average scores obtained on a 100-point grading system.

*The study results were processed* in Microsoft Excel using the online calculator Social Science Statistics. The Student’s t-test was used to compare indicators and determine the statistical validity and significance of the study results. The correlation between the scales was determined by calculating the Pearson correlation coefficient and comparing it with the table values from the Cheddock table.

*The study’s limitations* were that it was conducted only among Chinese students. It can be assumed that the socio-political structure of China and its influence on the education system, which determines the priority of ideological and political education in Chinese universities, has a significant impact on students’ motivation and evaluation of the prestige of the chosen specialty. At the same time, this factor is probably not so important in other countries. A comparative study of the motivational sphere of Chinese and international students and the impact of e-learning platforms on its dynamics is expected to be conducted in prospective studies.

*Ethical issues* in the research process were addressed by obtaining permission from university ethics commissions and the written informed consent of all respondents. The research process ensured the anonymity of the results, the confidentiality of the respondents’ data, and compliance with all other norms of bioethics.

## Results

The study results on students’ motivation to achieve learning success in the experimental and control groups in dynamics are shown in Table 1.

**Table 1:** Dynamics of motivation to achieve academic success among students of experimental and control groups (A, B and A1, B1 - preliminary and repeated studies, respectively)

GPA		Student’s t-test	*p*	GPA		Student’s t-test	*p*
A	A1			B	B1		
9.1 ± 0.4	15.3 ± 0.2	-25.22	< 0.05	9.2 ± 0.2	9.8 ± 0.5	-1.93	> 0.05

As can be seen from this table, in the preliminary study, students in both the experimental and control groups had an average level of motivation to achieve success, for which the groups were comparable with each other (the difference in indicators was statistically insignificant, t-value=-0.39, *p* > 0.05). Both groups showed increased motivation in the repeated study, but the control group’s indicator did not exceed the average level. It did not statistically differ from the initial one (9.2 ± 0.2 and 9.8 ± 0.5 points, *p* > 0.05). The students in the experimental group who were taught ideology and politics using electronic learning platforms, the dynamics of motivation to succeed in learning was more pronounced and amounted to 15.3 ± 0.2 points (high level) against 9.1 ± 0.4 points (average level), at *p* < 0.05. Table 2 presents the study results of the motivation dynamics of ideological and political learning at the university of the experimental and control groups students according to T. I. Ilyina’s method.

**Table 2:** Dynamics of the motives of ideological and political education at the university of students in the experimental and control groups (A, B and A1, B1 - preliminary and repeated studies, respectively) in line with T. I. Ilyina’s method

Scale	GPA		Student’s t-test	*p*	GPA		Student’s t-test	*p*
	A	A1			В	В1		
Acquisition of knowledge	8.5 ± 0.1	10.8 ± 0.4	-99.66	< 0.05	8.4 ± 0.2	8.8 ± 0.3	-1.92	> 0.05
Mastering a profession	7.7 ± 0.3	8.4 ± 0.2	-3.36	< 0.05	7.8 ± 0.2	8.0 ± 0.1	-1.55	> 0.05
Getting a diploma	8.2 ± 0.1	6.9 ± 0.1	15.92	< 0.05	8.0 ± 0.3	7.6 ± 0.2	1.92	> 0.05

In the preliminary study, there were no statistically significant differences between the indicators of the methodology scales of students from the experimental and control groups (*p* > 0.05). At the same time, the motive of obtaining a diploma prevailed. In the repeated study, the dynamics of motivation indicators of students in the control group were statistically insignificant (*p* > 0.05), while changes were more pronounced (*p* > 0.05) in the experimental group. Thus, in the initial study, the motive for obtaining a diploma was higher than that for mastering the profession. The second survey showed a statistically significant (*p* < 0.05) positive balance of indicators towards an increase and predominance of the motives of mastering a profession (from 7.7 ± 0.3 to 8.4 ± 0.2 points) and acquiring knowledge (from 8.5 ± 0.1 to 10.8 ± 0.4 points), with a decrease of the motive of obtaining a diploma (from 8.2 ± 0.1 to 6.9 ± 0.1 points). The data reflect the optimization of the ratio of motives. Table 3 presents the results to determine the dynamics of motives for exteriorization and interiorization of success in studying ideology and politics by experimental and control groups of students.

**Table 3:** Dynamics of exteriorization and interiorization motives for the success of studying the ideology and politics of students in the experimental and control groups (A, B and A1, B1 - preliminary and repeated studies, respectively)

Success motivation		GPA		Student’s t-test	*p*	GPA		Student’s t-test	*p*
Success type	Indicators	A	A1			В	В1		
Extrinsic	Standard of living	4.4 ± 0.1	4.5 ± 0.2	-0.18	> 0.05	4.3 ± 0.2	4.4 ± 0.3	-0.48	> 0.05
	Luck	4.0 ± 0.4	4.1 ± 0.3	-0.35	> 0.05	4.1 ± 0.2	4.1 ± 0.4	-1.58	> 0.05
	Recognition	4.5 ± 0.2	4.6 ± 0.1	-0.43	> 0.05	4.5 ± 0.1	4.5 ± 0.2	1.68	> 0.05
	Power	4.3 ± 0.2	4.4 ± 0.1	-0.77	> 0.05	4.2 ± 0.4	4.3 ± 0.6	-1.49	> 0.05
Intrinsic	Result of own activity	3.5 ± 0.1	4.2 ± 0.3	-3.83	< 0.05	3.5 ± 0.3	3.8 ± 0.2	-0.01	> 0.05
	Personal success	3.2 ± 0.4	4.2 ± 0.1	-4.20	< 0.05	3.3 ± 0.2	3.6 ± 0.3	-1.44	> 0.05
	Mental condition	3.1 ± 0.2	3.9 ± 0.4	-3.10	< 0.05	3.2 ± 0.3	3.4 ± 0.1	-1.10	> 0.05
	Overcoming obstacles	2.8 ± 0.5	3.6 ± 0.1	-2.71	< 0.05	2.7 ± 0.4	3.1 ± 0.2	-1.55	> 0.05
	Vocation	3.9 ± 0.3	4.5 ± 0.2	-2.71	< 0.05	4.0 ± 0.1	4.2 ± 0.3	-1.10	> 0.05

The preliminary study demonstrated no statistically considerable difference between the experimental and control groups’ extrinsic and intrinsic motivation indicators (*p* > 0.05). The level of extrinsic motivation in both groups was relatively high, but it underwent no statistically significant changes during the second study. Meanwhile, intrinsic motivation indicators showed a different picture. There was a slight, statistically insignificant growth (*p* > 0.05) in all subscales in the control group. At the same time, in the experimental group, there was a statistically reliable and significant (*p* < 0.05) increase in all subscales of the scale of intrinsic motivation for academic success: success as a result of one’s activity (from 3.5 ± 0.1 to 4.2 ± 0.3 points), personal success ( from 4.2 ± 0.3 to 4.2 ± 0.1 points), success as a mental condition (from 3.1 ± 0.2 to 3.9 ± 0.4 points), success as overcoming obstacles (from 2.8 ± 0.5 to 3.6 ± 0.1 points) and success as a vocation (from 3.9 ± 0.3 to 4.5 ± 0.2 points).

**Fig. 1:**
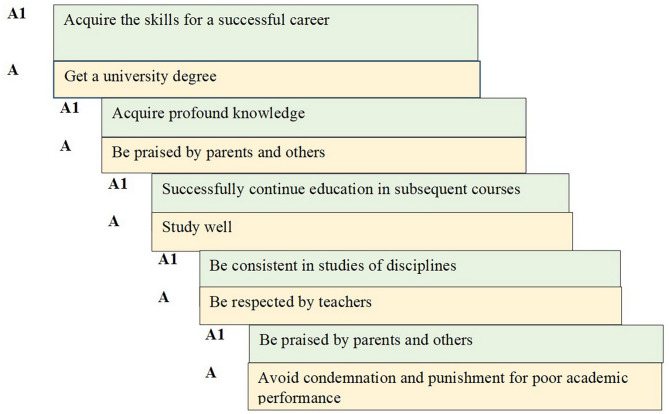
Dynamics of the hierarchy of motives for the ideological and political education of students in the experimental group (**A** - preliminary study, **A1** - repeated study)

The study of the dynamics of the hierarchy of motives of students receiving ideological and political education, according to the method of A. A. Rean, V. A. Yakunin, showed no significant changes in the preference of motives in the control group, in contrast to the experimental group as shown in Fig. 1.

In the preliminary study, among the motives of the educational activities of students receiving ideological and political education using e-learning platforms and in the control group, obtaining a diploma and parental approval, as well as the desire to study well and the fear of punishment and condemnation for poor study, prevailed. Meanwhile, during the repeated study, priority motives were significantly transformed in the experimental group. While the importance of the motive of the approval of parents and others remained, it fell to the last, fifth place in the rating. On the contrary, the first place was taken by the assurance of success in future professional activity, the second - the need to acquire profound knowledge, and the third - the need to successfully continue education in subsequent courses. At the same time, the motive of fear of condemnation and punishment for poor study has lost its significance. The motive of respect from teachers also ceased to bother students. Nevertheless, there was an awareness of the need to be consistent in studying the disciplines of the educational cycle as the basis for academic success. Table 4 shows the dynamics of the comparative assessment of the main motives for choosing a career by students in the experimental and control groups.

**Table 4:** Dynamics of the main motives for choosing a profession by students in the experimental and control groups (A, B and A1, B1 - preliminary and repeated studies, respectively)

Motive groups	GPA		Student’s t-test	*p*	GPA		Student’s t-test	*p*
	A	A1			В	В1		
Social	4.2 ± 0.1	4.3 ± 0.3	-0.55	> 0.05	4.1 ± 0.3	4.2 ± 0.2	-0.48	> 0.05
Moral	4.1 ± 0.4	4.2 ± 0.5	-0.27	> 0.05	4.0 ± 0.4	4.1 ± 0.3	-0.35	> 0.05
Aesthetic	2.7 ± 0.3	2.8 ± 0.2	-0.48	> 0.05	2.6 ± 0.4	2.9 ± 0.1	-1.26	> 0.05
Cognitive	3.6 ± 0.5	4.5 ± 0.1	-3.06	< 0.05	3.5 ± 0.4	3.8 ± 0.2	-1.16	> 0.05
Creative	2.5 ± 0.3	4.1 ± 0.2	-7.69	< 0.05	2.7 ± 0.5	2.9 ± 0.3	-0.40	> 0.05
Informative	3.0 ± 0.1	3.9 ± 0.4	-3.78	< 0.05	3.1 ± 0.2	3.3 ± 0.1	-1.55	> 0.05
Material	3.8 ± 0.3	4.0 ± 0.5	-0.59	> 0.05	3.7 ± 0.4	3.9 ± 0.3	-0.69	> 0.05
Prestigious	4.3 ± 0.2	4.4 ± 0.1	-0.77	> 0.05	4.2 ± 0.3	4.3 ± 0.1	-0.58	> 0.05
Utility	3.7 ± 0.2	3.8 ± 0.1	-0.77	> 0.05	3.6 ± 0.2	3.7 ± 0.1	-0.77	> 0.05

As can be seen from this table, the indicators of both groups in the preliminary study were comparable, and there was no statistically significant difference between them (*p* > 0.05). During the second study, the indicators of the importance of the motives for choosing a profession by the students in the control group underwent no substantial changes (*p* > 0.05). In the experimental group, the dynamics of the indicators of the importance of social, moral, aesthetic, material, prestigious and utilitarian motives were also not statistically significant. At the same time, in the experimental group, statistically reliably (*p* < 0.05) significantly increased the indicators of the significance of such motives of a career choice as cognitive (from 3.6 ± 0.5 to 4.5 ± 0.1 points), creative (from 2.5 ± 0.3 to 4.1 ± 0.2 points) and informative (from 3.0 ± 0.1 to 3.9 ± 0.4 points).

The study’s results in the dynamics of the levels and types of motivation for ideological and political education of the students in the experimental group who studied using e-learning platforms and those in the control group who did not use digital technologies in the experiment are presented in Table 5.

**Table 5:** Dynamics of levels and types of motivation for ideological and political education of students in the experimental and control groups (A, B and A1, B1 - preliminary and repeated studies, respectively)

Motive type	Motive level	GPA		Student’s t-test	*p*	GPA		Student’s t-test	*p*
		A	A1			В	В1		
Cognitive	Broad	2.5 ± 0.2	3.5 ± 0.4	-3.87	< 0.05	2.4 ± 0.3	2.7 ± 0.2	-1.44	> 0.05
	Narrow	2.6 ± 0.1	3.6 ± 0.3	-5.48	< 0.05	2.5 ± 0.2	2.8 ± 0.3	-1.44	> 0.05
Social	Broad	2.8 ± 0.4	3.4 ± 0.1	-2.52	< 0.05	2.8 ± 0.5	3.0 ± 0.4	-0.54	> 0.05
	Narrow	2.7 ± 0.3	3.4 ± 0.2	-2.86	< 0.05	2.7 ± 0.2	2.9 ± 0.1	-1.55	> 0.05

Initially, all types and levels of motives for the ideological and political education of students in the experimental group were comparable and did not differ considerably (*p* > 0.05). During the repeated study, there were no significant changes in motivation indicators in the control group (*p* > 0.05). Meanwhile, the experimental group saw a statistically significant (*p* < 0.05) rise in indicators on all scales of the methodology to a level above the average (broad cognitive motives - from 2.5 ± 0.2 to 3.5 ± 0.4 points, narrow cognitive motives - from 2.6 ± 0.1 to 3.6 ± 0.3 points, broad social motives - from 2.8 ± 0.4 to 3.4 ± 0.1 points, and narrow social motives - from 2.7 ± 0.3 to 2.7 ± 0.3 points).

Between the scales of methods determined by cognitive motives, a high close direct correlation was found (Pearson’s correlation coefficient rxy = 0.88 at *p* < 0.05), while with the scales of social reasons, the correlation was also direct but moderately pronounced (rxy = 0.59 at *p* < 0.05).

The control test of the students’ assimilation of the theoretical material showed that the basic level of knowledge of the respondents in the experimental and control groups was practically the same during the initial study (68.3 ± 1.4 and 70.0 ± 1.2 points, t-value=-1.60, *p* > 0.05). The repeated testing revealed a statistically significant (*p* < 0.05) growth in both study groups’ theoretical knowledge of educational material. In the control group, the average knowledge assessment score during repeated testing was 76.1 ± 1.5 points (t-value=-5.50, *p* < 0.05), and in the experimental group, it was 82.4 ± 2.0 points (t-value=-10.0, *p* < 0.05). Obviously, the performance of students in the experimental group, who received ideological and political education using e-learning platforms, increased more significantly against the control group, which is also confirmed by a statistically reliable and significant difference between these indicators (t-value=-4.36, *p* < 0.05).

## Discussion

Motivation for learning is no less significant factor influencing students’ academic performance than the level of innate abilities and the development of cognitive interests. A comparison of the experimental and control groups shows that ideological and political education using the MOODLE electronic learning platform contributes to a statistically significant increase in students’ motivation for learning activities. In contrast, there is no significant change in motivation indicators with traditional learning.

It is worth noting that the use of e-learning platforms is a means of encouraging students to succeed in learning activities, which is supported by the results of our study regarding the use of the MOODLE platform for ideological and political learning of Chinese students. At the same time, there is a positive transformation of educational motives toward their greater adequacy. The initial dominance of the motivation for education to obtain a diploma is interpreted by researchers [42] as a reflection of the personal desire to document success. The need for it is associated with fulfilling obligations to parents and society. This assumption is supported by the fact that in the initial study of the hierarchy of motives, the desire to gain approval from parents and the environment ranked second after the motive to obtain a diploma. As students who study through e-learning platforms become familiar with their future profession, the hierarchy of motives changes, with the predominance of achieving professional success, which necessitates acquiring deep and solid knowledge. Students begin to realize the importance of education for future professional success, think ahead, and plan for further learning. Greater involvement and immersion in mastering the future profession in education using e-learning platforms make it preferable for students compared to traditional classroom learning, as evidenced by data from scientific literature sources [14].

The separation of intrinsic and extrinsic motivation is considered in the scientific literature [42] as the formation of the desire to get a profession under the influence of external factors (social, material, and psychological) or personal interests and needs. During the study, both in the control and experimental group, the level of external motivation of students (desire for recognition, power, material well-being) was initially high enough and did not undergo statistically significant changes in the dynamics. Intrinsic motivation in the control group also did not change significantly in the repeated study, while in the experimental one, it statistically notably increased on all scales of the method. This fact gives grounds to assert that ideological and political education using the MOODLE e-learning platform contributes to students’ readiness to overcome obstacles and intensify their focus on achieving success and awareness of their potential to achieve success in professional self-realization. At the same time, one can agree with the opinion of researchers [42] about the factor of unconscious social coercion inherent in Chinese students to achieve high results. It is confirmed in the study by identifying the high significance of the motivation of approval, respect, and avoidance of condemnation and punishment during the first test. It should be noted that there was a significant correction of the hierarchy of motives in the experimental group, which indicates the advantages of learning using the electronic educational platform MOODLE compared to the traditional one in relation to the motivational component of the educational process. The positive dynamics of motivation in the experimental group also contributed to greater learning efficiency, which was reflected in a more significant increase in the level of assimilation of theoretical knowledge and, accordingly, in the academic performance of students compared to the control group who studied traditionally. Thus, the current research results confirm the data from scientific literature sources [18] on the positive impact of e-learning platforms on improving students’ educational competencies and boosting their personal and professional growth. An increase in cognitive, creative and informative learning motives in the experimental group manifests this. Developing a cognitive motive indicates the formation of sustainable professional competencies among students and a rise in their readiness to independently solve professional problems [45]. Thus, the use of the MOODLE e-learning platform creates psychological conditions that are more suitable compared to traditional learning. They increase motivation to improve in the chosen profession [23, 45]. The conditions also contribute to the independence and awareness of the motives for achieving educational and professional success and normalizing the hierarchy of motivations. Considering the fundamental importance of motivation in achieving academic success among students [19], it can be argued that the results of the study convincingly indicate the benefits of using the MOODLE e-learning platform in ideological and political education.

## Conclusions

The present research demonstrated the importance of ideological and political education involving e-learning platforms to increase students’ motivation for learning activities. A statistically significant positive balance of indicators was observed in the direction of increase and predominance of motives for mastering a profession and acquiring knowledge. Meanwhile, a decrease in the motive of obtaining a diploma was observed, which reflects the optimization of the ratio of motives in ideological and political education using e-learning platforms.

A statistically significant positive dynamics of internal motivation for success in studies was revealed. A positive transformation of the hierarchy of motives in ideological and political education using e-learning platforms was also shown.

In the course of ideological and political education using e-learning platforms, a statistically significant increase in the significance of cognitive, creative and informative motives for choosing a profession was determined.

Yet, ideological and political education through e-learning platforms contributes to a more significant increase in students’ academic performance than traditional classroom learning.

Thus, as a result of the study, the significance of the motivational component in achieving the success of ideological and political education and the impact of using e-learning platforms on students’ motivation has been theoretically substantiated. It has been confirmed that applying e-learning platforms in ideological and political education helps step up student motivation and academic performance.

The present findings are useful in enhancing the effectiveness of ideological and political education in Chinese universities. They also interest the international scientific and pedagogical community concerning a global multicultural educational environment.

Future research is expected to focus on a comparative study of the motivational sphere of Chinese and international students and its impact on the dynamics of using e-learning platforms.

## Data Availability

Data will be available from the corresponding author upon reasonable request.
